# Relationship between Sitting Time and Insulin Resistance in 6931 U.S. Adults: The Mediating Role of Abdominal Adiposity

**DOI:** 10.1155/2023/5015572

**Published:** 2023-05-24

**Authors:** Kayla M. Parker, Larry A. Tucker, Bruce W. Bailey, Lance E. Davidson

**Affiliations:** Department of Exercise Sciences, Brigham Young University, Provo, Utah 84602, USA

## Abstract

This cross-sectional investigation examined the relationship between sitting time and insulin resistance in 6931 U.S. adults. The mediating effects of several covariates were evaluated. Self-reported sitting time, measured in minutes per day, was the exposure variable. Insulin resistance (IR), indexed using the natural log of the homeostatic model assessment of insulin resistance (L-HOMA-IR), was the outcome variable. This study used data collected from the 2011–2018 National Health and Nutrition Examination Survey (NHANES). Results showed a strong, positive, dose-response association between sitting time and insulin resistance after adjusting for age, sex, race, and year of assessment (*F* = 12.6, *p* < 0.0001). Across the sitting time tertiles (low, moderate, and high), the L-HOMA-IR means (±SE) each differed from each other (0.37 ± 0.008, 0.40 ± 0.012, and 0.43 ± 0.012). Further controlling for cigarette smoking and physical activity did not alter the significance of the relationship. Adding body mass index (BMI) to the demographic covariates weakened the relationship, but it remained significant. However, the association was no longer significant after adjusting for the demographic covariates and waist circumference (*F* = 1.1, *p* = 0.3349). None of the L-HOMA-IR means (±SE) differed from each other (0.40 ± 0.007, 0.41 ± 0.009, and 0.41 ± 0.008). Overall, waist circumference was a powerful mediating variable between sitting time and insulin resistance. Apparently, time spent sitting is a powerful predictor of IR. However, much of the association between sitting time and IR is a function of differences in waist size. As a strong measure of abdominal adiposity and a significant predictor of multiple metabolic diseases, managing waist size is a health practice to consider when insulin resistance is a concern.

## 1. Introduction

Sedentary behaviors have become more common in recent decades with the increase in desk jobs, advancements in transportation, and rise in computer use and screen time. Sedentary behavior is “any waking behavior characterized by an energy expenditure ≤ 1.5 metabolic equivalents (METS), while in a sitting, reclining, or lying posture” [1]. This is not to be confused with physical inactivity, which implies a failure to reach physical activity recommendations [1]. Thus, active individuals who meet physical activity recommendations may be simultaneously considered sedentary if they also spend most of the day sitting [1].

There is an abundance of scientific evidence supporting the effects of physical activity, or the lack thereof, on disease risk. However, recent emphasis has been placed on studying the specific effects of sedentary behavior on various cardiometabolic risk factors and diseases [1]. One such risk factor is insulin resistance. Insulin resistance is characterized by an impaired response of the body to insulin, resulting in an impaired peripheral tissue glucose uptake [2]. Impaired glucose uptake can lead to elevated blood glucose and development of type 2 diabetes mellitus, if left untreated [2].

Substantial evidence suggests a positive correlation between sedentary time and insulin resistance [3–9]. Research has further indicated that there are several variables that may mediate this relationship, such as age, sex, race, body mass index (BMI), and physical activity. While a few studies have included physical activity and BMI as covariates, almost none have included a measure of abdominal adiposity. Yet, abdominal obesity is highly related to insulin resistance [10–12]. Among the few investigations that have controlled for differences in abdominal adiposity, sample sizes have been relatively small, and results have been inconsistent.

Waist circumference is a good indicator of obesity, especially abdominal adiposity, which is a significant predictor of morbidity and mortality [13]. Research shows that measures of abdominal adiposity are stronger predictors of all-cause mortality [14], cardiovascular disease mortality [15], diabetes mellitus [16], and unfavorable metabolic profiles [17] than BMI. In other words, the distribution of body fat may have more health implications than total body fat mass. This highlights the importance of better understanding the mediating roles of BMI and waist circumference in the relationship between sedentary time and insulin resistance.

This study focused on determining the relationship between sedentary time and insulin resistance in a random sample of 6931 U.S. adults. It also examined how this association was adjusted by demographic factors, cigarette smoking, physical activity, BMI, and waist circumference, particularly the latter two.

## 2. Methods

### 2.1. Study Design

This cross-sectional study used data collected from the National Health and Nutrition Examination Survey (NHANES), a large-scale, ongoing investigation focused on assessing human health and nutrition among the Unites States population. NHANES is a research program directed by the National Center for Health Statistics (NCHS). As part of the survey, trained technicians perform physical examinations, take blood samples, and conduct comprehensive interviews on randomly selected individuals in two-year cycles. The current study utilized NHANES data from 2011 to 2018 because the COVID pandemic prevented data from being collected from 2019 to 2020 and after.

### 2.2. Subjects

A total of 6931 U.S. adults aged 20–79 years were included in the present study. Participants were selected using a multistage, random-sampling strategy. To be representative of the U.S. population, census data were used to select subsamples from randomly selected counties, blocks, and dwelling units [18]. Written informed consent was submitted by each subject prior to participation, and the collection of data was approved by the Ethics Review Board (ERB) for the NCHS [19].

Only participants with complete data were included in the investigation. Across the 8 years of data collection (2011–2018), a total of 39,156 women, men, and children, including newborns, were surveyed by NHANES. From the total sample, a subsample of 11,223 individuals aged 12 years and older was randomly selected by NHANES to have a blood sample collected. Additionally, to maximize confidentiality, adults > 80 years old were each reported by NHANES to be age 80. Given the intentional truncated ages for those > 80, the sample was delimited to individuals 20–79 years old, decreasing the sample by 2499, leaving 8724 in the subsample. There were 81 pregnant women who were not included, reducing the sample to 8643. A total of 230 adults were not included because they were underweight (BMI < 18.5 kg/m^2^), resulting in a sample of 8413. Adults who were diabetic, who had elevated fasting blood glucose levels (> 126 mg/dL), or who were taking medication to control their blood glucose levels were not included in the analyses (*n* = 1271), leaving 7142 in the subsample. There were 210 individuals who had missing waist measurements and 1 without cigarette-smoking data, leaving 6931 in the final sample.

### 2.3. Sitting Time

The predictor variable in this study was sitting time, indicating the degree of sedentary behavior. Sitting time has been linked with many chronic diseases and increased risk of mortality [20, 21]. With modern technologies and an increasing number of desk jobs, it is not uncommon for individuals to spend a large portion of their day in sedentary behaviors [1]. Participants in this study were asked the following question: “How much time do you usually spend sitting or reclining on a typical day?” [18].

### 2.4. Insulin Resistance

Insulin is a peptide hormone produced by the pancreatic beta cells that mediates the glucose uptake into peripheral tissues [22]. In cases of insulin resistance, the body's cells do not properly respond to insulin, resulting in impaired glucose uptake and elevated blood glucose [22]. Insulin resistance, the criterion variable, was determined using the homeostatic model assessment for insulin resistance (HOMA-IR). Because the distribution was skewed, the log of HOMA-IR was used (L-HOMA-IR).

According to NHANES, during a private examination session, trained nurses collected a fasting blood sample from participants. A minimum of 9 hours of fasting was required. Fasting plasma glucose [23] and insulin [24] levels were recorded and employed to calculate HOMA-IR using the following formula: fasting insulin (*μ*U/mL) × fasting glucose (mg/dL)/405 [25]. Higher HOMA-IR values indicate greater insulin resistance.

### 2.5. Covariates

NHANES used the following 6 categories to classify race: non-Hispanic (NH) White, NH Black, NH Asian, other Hispanic, Mexican American, or other/multiracial. This study statistically controlled for differences in race.

The year of assessment was documented and controlled during statistical analyses because data were collected across 8 different years (2011–2018).

Because cigarette smokers tend to be leaner than nonsmokers, smoking was also included as a covariate [26]. Smoking was self-reported and recorded as the average number of cigarettes smoked per day in the past 30 days [27]. Because the cigarette-smoking variable was highly skewed, a categorical variable was used. Specifically, three categories were formed: (1) nonsmokers, (2) those who reported smoking less than one pack per day, and (3) those who reported smoking a pack or more per day.

Another covariate was physical activity level, quantified as the sum of moderate to vigorous physical activity (MVPA) in minutes per week. This information was collected via interview. According to NHANES, moderate physical activity was characterized as activity leading to slightly elevated heart rate or breathing, for example, walking or casual cycling. Vigorous physical activity was described as activity leading to significant elevations in heart rate or breathing. Examples include walking on a steep incline, jogging, running, or lifting heavy loads. Physical activity appears to be consistently related to levels of insulin resistance [28]. Research also suggests that physical activity may be a mediating variable in the association between sitting time and HOMA-IR [29] due to its role in increasing insulin-mediated glucose uptake and glucose transporter type 4 (GLUT4) expression [28].

Evidence from the literature has consistently shown a positive linear relationship between BMI and insulin resistance [30]. Body weight was assessed using a digital scale. The scale was custom made for NHANES. The scale was built into the exam room floor. This high-performance instrument was linked directly to the anthropometry computer application to minimize recording errors. Subjects wore underwear and a disposable paper gown for the weigh-in. A fixed stadiometer with a mobile headboard was used to measure subjects' height while standing [31]. Like the digital weight scale, the stadiometer was also custom built. A digital measurement device was connected to the acrylic head piece. It interfaced directly with the anthropometry computer application. A 15-centimeter plastic ruler was used to correct stature measurements when subjects had hair styles that interfered with the stadiometer headpiece placement. The recorder entered the height correction factor into the application, which automatically calculated an adjusted height value. An 80-centimeter metal rod was used to calibrate the stadiometer on a weekly basis.

Body mass index (BMI) was subsequently calculated for each subject as follows: weight (kg)/square of height (m^2^).

Abdominal obesity was indexed using waist circumference measurements. This study controlled for waist circumference due to its strong association with insulin resistance [30]. To acquire the waist measurement, subjects were instructed to cross their arms and place their hands on opposite shoulders, as if they were giving themselves a hug. Two trained specialists were used to obtain each waist circumference measurement, an examiner and a recorder. The examiner stood on the participant's right side. The examiner palpated the hip area to locate the right ilium of the pelvis. With a cosmetic pencil, the examiner drew a horizontal line just above the uppermost lateral border of the right ilium. This mark was then crossed at the midaxillary line, which extends from the armpit down the side of the torso. If it was difficult to find the iliac crest, such as on participants with larger waists, then the examiner began inferior to the midaxillary line (toward the subject's front) and palpated the ilium upward to the midaxillary line until the uppermost part of the bone was found. The measuring tape was then horizontally positioned around the waist at the level of the measurement mark. A wall mirror was used to ensure proper tape alignment. While the examiner remained on the subject's right side, the recorder moved around to the subject's left side to check the placement of the tape. Both checked that the tape sat parallel to the floor and was snug but did not compress the skin. The zero end of the tape was positioned below the section containing the measurement value. The measurement was taken to the nearest 0.1 cm at the end of the subject's normal exhalation [31]. The measuring tape was a Lufkin Executive (Missouri City, Texas, USA).

### 2.6. Statistical Analysis

To select study participants, NHANES utilized a unique multistage sampling strategy. For results to be generalizable to all noninstitutionalized adults in the U.S., individual sample weights, in addition to randomly selected strata and clusters, were utilized in the statistical analyses.

While a large sample size of 6931 participants would produce excellent statistical power under normal circumstances, the multilevel sampling strategy used by NHANES substantially reduces statistical power. Due to nesting, the degrees of freedom (df) in the denominator were not 6931, but rather 62, calculated by subtracting the number of strata (59) from the number of clusters (121).

A total of six models were employed. The first model adjusted for differences in age, sex, race, and year of assessment. The second model controlled for these variables and cigarette smoking. The third model controlled for all the model 1 covariates plus physical activity. The fourth model adjusted for all the model 1 covariates and also BMI. The fifth model controlled for the model 1 covariates plus waist circumference. The sixth model adjusted for all the covariates simultaneously.

Because the waist circumference covariate had a strong mediating impact on the sitting time and insulin resistance relationship, effect modification was tested. Specifically, the association between sitting time and insulin resistance was measured across sex-specific tertiles of waist circumference.

A mediation analysis was conducted showing the relationship between sitting time and waist circumference, between waist circumference and insulin resistance, and between sitting time and insulin resistance. Finally, the analysis showed the relationship between sitting time and insulin resistance after adjusting for differences in waist circumference. For each step of the mediation analysis, the demographic covariates, age, sex, race, and year of assessment, were controlled statistically.

Sitting time was divided into tertiles because it had a skewed distribution. However, because many of the tertile cut points included a large number of participants, *N* differed across the 3 sitting time categories (low: *N* = 2483, moderate: *N* = 1868, and high: *N* = 2580). These frequencies are listed as weighted values due to person-level weighted adjustments used by NHANES.

HOMA-IR was expressed as a continuous variable. Mean differences in HOMA-IR were compared across the three categories of sitting time using one-way analysis of variance (ANOVA) and linear regression. Means were adjusted for differences in the covariates using partial correlation and the LSmeans procedure. The natural log of the HOMA-IR values (L-HOMA-IR) was taken, so the distribution was no longer skewed. SAS version 9.4 (SAS Institute, Inc., Cary, NC, USA) was utilized to run the statistical analyses. All *p* values were two-sided, and alpha was established as <0.05 for statistical significance.

## 3. Results

This study included a total of 6931 subjects, representative of the U.S. adult population. Average age (±SE) was 45.1 (±0.3) years. Mean sitting time was 376.1 (±4.3) minutes per day. The average HOMA-IR was 3.19 (±0.06), and the average L-HOMA-IR was 0.37 (±0.01). Furthermore, mean minutes of MVPA per week were 165.3 (±5.5). Average waist circumference (cm) and BMI were 99.1 (±0.36) and 29.1 (±0.15), respectively.  Table 1 displays a summary of values across percentiles for each of the continuous variables used in this investigation.

The categorical variables in this study were sex, race, cigarette smoking, and sitting time. Of the 6931 subjects, 3546 were women, and 3385 were men. The NHANES race categories and respective percentages for the total sample were as follows: non-Hispanic White (64.8%), non-Hispanic Black (10.9%), Mexican American (9.1%), other Hispanic (6.6%), non-Hispanic Asian (5.3%), and other or multiracial (3.3%). Because the tertile cut points included a large number of participants, *N* differed across the 3 sitting time categories (low: *N* = 2483, moderate: *N* = 1868, and high: *N* = 2580). For smoking, 80.7% reported no cigarette smoking, 13.9% reported smoking less than 1 pack per day, and 5.4% indicated that they smoked a pack or more per day.

Table 2 compares mean L-HOMA-IR across the low, moderate, and high categories of sitting time. Model 1 controlled for the demographic covariates only (age, sex, race, and year of assessment). L-HOMA-IR differed significantly across all three categories of sitting time in a dose-response fashion (*F* = 12.6 and *p* < 0.0001). Specifically, subjects in the highest category of sitting time had the greatest L-HOMA-IR, followed by those in the moderate group, followed by those in the low group. Model 2 controlled for the demographic covariates plus cigarette smoking. Again, L-HOMA-IR differed significantly across the three categories of sitting time (*F* = 12.0 and *p* < 0.0001) in the same dose-response manner as model 1.

In Table 2, model 3 controlled for the demographic variables and physical activity, measured in minutes of MVPA per week. Similar to models 1 and 2, model 3 also showed that each L-HOMA-IR mean differed significantly from other means in a dose-response manner (*F* = 10.5 and *p* < 0.0001). Model 4 controlled for the demographic variables and BMI. After adding BMI to the model, the relationship between sitting time and L-HOMA-IR was still statistically significant; however, it was weakened (*F* = 3.7 and *p* = 0.0293). Unlike the first three models, mean L-HOMA-IR only differed between the low and high sitting time categories.

Model 5 in Table 2 controlled for the demographic factors and waist circumference. Results showed that the relationship between sitting time and L-HOMA-IR was no longer significant after adding waist circumference to the model (*F* = 1.1 and *p* = 0.3349). No differences in mean L-HOMA-IR existed between the low, moderate, and high categories of sitting time.

**Figure 1:**
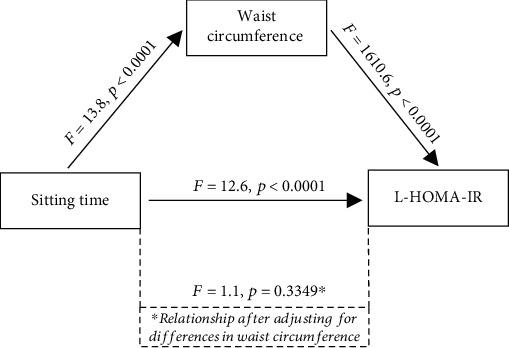
Mediation analysis results.

Figure 1 displays the step-by-step results of the mediation analysis. The evaluation showed that the direct association between sitting time (tertiles) and L-HOMA-IR was significant (*F* = 12.6 and *p* < 000.1). L-HOMA-IR differed significantly across each tertile of sitting time in a dose-response pattern. Step 2 of the mediation analysis showed that sitting time (tertiles) was also a significant predictor of waist circumference, a potential mediating factor. Specifically, after adjusting for the demographic variables, mean waist circumferences differed significantly across the sitting time tertiles (*F* = 13.8 and *p* < 0.0001), in a dose-response manner (mean ± SE): low (96.4 ± 0.43), moderate (98.4 ± 0.64), and high (100.1 ± 0.53). Step 3 showed that the waist circumference was a strong predictor of L-HOMA-IR after adjusting for differences in the demographic covariates (*F* = 1610.6 and *p* < 0.0001). Finally, step 4 showed that sitting time (tertiles) was no longer associated with L-HOMA-IR after controlling for differences in the demographic covariates and waist circumference. In short, the association between sitting time and L-HOMA-IR was eliminated (*F* = 1.1 and *p* = 0.3349).

Because of the strong mediating influence of waist circumference on the association between sitting time (tertiles) and L-HOMA-IR, effect modification was tested. Specifically, the relationship between sitting time (tertiles) and L-HOMA-IR was evaluated across sex-specific tertiles of waist circumference. As shown in Table 3, after adjusting for differences in the covariates, L-HOMA-IR did not differ across any of the sitting time categories within any of the sex-specific waist circumference tertiles.

The correlation between waist circumference and BMI was strong after adjusting for differences in the demographic covariates: age, sex, race, and year of assessment (*R*^2^ = 0.80 and *p* < 0.0001). However, the relationship between sitting time (tertiles) and L-HOMA-IR remained significant after adjusting for differences in the demographic variables and BMI (Table 2, model 4: *F* = 3.7 and *p* = 0.0293). However, the association was abolished when waist circumference was controlled instead of BMI (Table 2, model 5: *F* = 1.1 and *p* < 0.3349).

## 4. Discussion

The main objective of the present study was to determine the relationship between sitting time and insulin resistance in 6931 adults representing the U.S. population. Another aim was to determine the extent to which age, sex, race, and year of assessment influenced the association between sitting time and insulin resistance. The final purpose was to evaluate the mediating roles of cigarette smoking, physical activity, BMI, and waist circumference in the relationship between sitting time and insulin resistance, particularly BMI and waist circumference.

Findings showed that there was a significant, linear association between sitting time, measured in minutes per day, and insulin resistance, indexed by L-HOMA-IR. Those in the lowest category of sitting time had the lowest L-HOMA-IR values, followed by those in the moderate category, then the highest category of sitting time. Statistically controlling for differences in age, sex, race, and year of assessment did not alter the significance of the relationship. The association remained unchanged after separately adding cigarette smoking and physical activity to the model. Adjusting for BMI, in addition to the demographic factors, weakened but did not nullify the relationship between sitting time and insulin resistance. However, results revealed that the association was no longer significant after including waist circumference in the model with the demographic variables.

Stated another way, waist circumference completely mediated the relationship between sitting time and insulin resistance. It appears that BMI partially mediates the sitting time and insulin resistance relationship. However, the mediating role of the waist circumference appears to be much more powerful than that of BMI. In a large, national sample, after adjusting for differences in abdominal adiposity, there was no association between sitting time and insulin resistance, as shown in Table 2 (model 5) and Figure 1.

Because differences in waist circumference had a powerful nullifying influence on the sitting time and insulin resistance association, the impact of different waist sizes was further evaluated using effect modification. Specifically, the relationship was assessed across sex-specific tertiles of waist circumference. As shown in Table 3, there was no relationship between sitting time and insulin resistance within any of the waist circumference tertiles. This finding further supports the mediating role of abdominal adiposity. Apparently, it is the variation across the full spectrum of waist sizes, from small to large, that influences the sitting time and insulin resistance connection. With differences in abdominal adiposity delimited to only small, only moderate, or only large waists, the link between sitting time and insulin resistance is broken.

Results showed that U.S. adults sit for an average of 376.1 minutes per day or approximately 6.3 hours. Data from NHANES further suggests that adults spend over half of their waking time in sedentary behaviors [32]. The 2018 Advisory Committee for the *Physical Activity Guidelines for Americans: 2^nd^ Edition* concluded that there is significant evidence to support the association between sedentary behavior and risk of chronic disease and all-cause mortality [32]. However, they determined that research was insufficient to provide specific recommendations regarding daily sedentary time, given that time spent in MVPA outside of sedentary time may influence the degree of risk [32]. Still, risk of all-cause mortality and cardiovascular disease appears to rise with increased sedentary time, and individuals are encouraged to limit sedentary behaviors [32].

A number of other cross-sectional studies have revealed similar positive associations between sitting time and insulin resistance. Many of these studies have controlled for demographic factors and have also evaluated the mediating influence of physical activity, yet few have controlled for BMI, and almost none have controlled for waist circumference or another measure of abdominal adiposity.

For example, Staiano et al. examined a sample of 4560 adults taken from the 2007–2010 National Health and Nutrition Examination Survey (NHANES) [9]. The number of hours spent sitting was self-reported, and HOMA-IR was assessed, along with other cardiometabolic risk factors [9]. Multivariable linear regression analyses showed that sitting time was significantly related to HOMA-IR in both men and women [9]. Similar to the present study, this relationship was not attenuated after controlling for MVPA [9]. However, the Staiano investigation did not control for BMI or waist circumference [9].

Kim et al. conducted an analysis using self-reported data from the 2015 Korean National Health and Nutrition Examination Survey [4]. Reported sedentary time was split into four groups by quartiles [4]. Individuals in the highest quartile (reporting ≥10 h/day sedentary time) had 1.4 times greater odds of having high insulin resistance (HOMA − IR > 1.6) compared to individuals in the lowest quartile [4]. Controlling for MVPA and BMI did not significantly alter the relationship between sedentary time and HOMA-IR. No measure of abdominal adiposity was used as a covariate, however [4].

In another study conducted by García-Hermoso et al., 1122 adults wore Actigraph accelerometers to objectively measure sedentary time over the course of seven days [29]. Subjects were stratified into three categories of sedentary time (low, medium, and high) [29]. Results showed that participants in the low and medium sedentary time groups had significantly lower HOMA-IR values compared to those in the high sedentary group [29]. In contrast to the present investigation, the García-Hermoso study did not adjust for differences in BMI or waist circumference [29]. Furthermore, after adjusting for MVPA, the association between sedentary time and HOMA-IR was no longer significant, suggesting that the relationship was influenced by MVPA [29].

There are also a number of prospective studies in the literature that support the association between sitting time and insulin resistance, showing mixed results regarding the mediating effects of physical activity, BMI, and waist circumference.

In an evaluation of 2027 young adults in the CARDIA study, Barone et al. observed the correlation between sedentary time and metabolic health over the course of five years [33]. Sedentary time was assessed using accelerometers, and HOMA-IR was measured at baseline and after 5 years [33]. While no 5-year differences were observed in HOMA-IR or fasting insulin, cross-sectional analyses showed that every hour-per-day increase of sedentary time was correlated with a 3% greater HOMA-IR and fasting insulin [33]. Controlling for MVPA and BMI in cross-sectional analyses weakened the relationship between sedentary time and fasting insulin and HOMA-IR, but it remained statistically significant [33]. As with other studies, waist circumference was not statistically controlled [33].

A similar investigation performed by Cooper et al. examined cross-sectional and longitudinal relationships between objectively measured sedentary time and HOMA-IR [34]. The subjects were recently diagnosed type 2 diabetes patients. In cross-sectional analyses, sedentary time was significantly related to HOMA-IR after adjustment for MVPA (*β* = 0.42 and *p* = 0.004); however, the relationship was no longer significant after also controlling for waist circumference (*β* = 0.23 and *p* = 0.091) [34]. Longitudinal analyses revealed that sedentary time at baseline also correlated with HOMA-IR at the 6-month follow-up after adjustment for demographics, MVPA, and waist circumference (*β* = 0.49 and *p* = 0.02) [34]. BMI was not listed as a covariate [34].

In a longitudinal study by Helmerhorst et al., researchers followed 376 adults for 5.6 years [35]. Helmerhorst et al. assessed physical activity and sedentary time objectively using heart rate monitoring over the course of 4 days [35]. Helmerhorst et al. also used fasting plasma insulin as a surrogate measure of insulin resistance [35], whereas the present study utilized HOMA-IR.

Helmerhorst et al. found a significant, weak, and positive correlation between baseline sedentary time and fasting plasma insulin at the 5.6-year follow-up (*r*^2^ = 0.021 and *p* = 0.005) [35]. Controlling for covariates, including age, sex, fat mass, baseline fasting insulin, smoking status, and duration of follow-up, weakened the relationship, but it remained statistically significant (*r*^2^ = 0.439 and *p* = 0.015) [35]. In additional analyses, the covariate fat mass was replaced by waist circumference or body fat percentage [35]. Unlike the present study, results remained unchanged with these substitutions. Further controlling for MVPA also produced virtually no change in the magnitude of the relationship (*r*^2^ = 0.441 and *p* = 0.009) [35].

While a majority of studies support the general findings of the present study, a couple have produced opposing results. A cross-sectional study by McGuire and Ross evaluated the association between physical activity behaviors and HOMA-IR in 135 inactive adults with abdominal obesity [36]. Contrary to previous studies, sedentary time was not significantly related to HOMA-IR (*p* = 0.46), and the association remained insignificant after controlling for waist circumference [36].

A longitudinal study conducted by Ekelund et al. also found no relationship between sedentary time and insulin resistance [37]. Activity level and sedentary time were measured via accelerometry at baseline and one-year follow-up in 192 adults with a family history of type 2 diabetes [37]. Both cross-sectional analyses at baseline and prospective analyses at follow-up showed that objectively measured sedentary time was not significantly related to HOMA-IR (*p* = 0.42 and *p* = 0.39, respectively) [37]. The model was adjusted for demographic variables and waist circumference, but the adjustment did not alter the significance of the relationship [37].

While not completely elucidated, there are several mechanisms that could explain the findings of the present study. The literature indicates that there are many negative consequences of sitting for long durations that may link sedentary behavior to insulin resistance. First, sitting may take the place of physical activity that is crucial for maintaining healthy metabolic processes in the body [38, 39]. Physiologically, lack of physical activity may be tied to decreased glucose uptake and subsequent elevated blood glucose and insulin action dysfunction, both of which contribute to insulin resistance [29]. In the present study, MVPA did not mediate the relationship between sitting time and insulin resistance. Because most U.S. adults reported little or no regular MVPA, it is possible there was insufficient activity to offset the negative health consequences of sitting [9].

Another possible explanation for these results is that those who engage in large amounts of sedentary behavior may also exhibit other unhealthy lifestyle behaviors (not controlled in this study) that contribute to metabolic dysfunction, such as an unhealthy diet [40, 41], excess alcohol consumption [42], or chronic stress [43]. Additionally, snacking and overeating are more likely to occur during sedentary periods [44]. Given that abdominal obesity and BMI are highly related to insulin resistance [10–12, 45], excess adiposity may be a linking factor between sitting and insulin resistance [44, 46, 47]. This idea is supported by results of the current study, which showed that waist circumference and, to a lesser extent, BMI were the primary variables accounting for the observed association between sitting time and insulin resistance.

The powerful mediating influence of waist circumference may be explained by its independent associations with both sitting time and insulin resistance. Studies in the literature have repeatedly shown a significant correlation between increased sedentary time and excess abdominal adiposity [48–51]. As discussed, overnutrition and lack of physical activity resulting from sedentary behaviors are likely links [38, 39]. However, the reverse association is also supported. Obese individuals may be less physically mobile with a greater propensity for sitting [52, 53].

The association between abdominal adiposity and insulin resistance is also well-established [10–12, 45]. Research supports a positive relationship between overall adiposity (i.e., BMI) and insulin resistance; however, the location of fat depots is a significant determinant of the degree of association [45]. While subcutaneous fat may, under some circumstances, contribute to metabolic dysfunction, abdominal fat is much more strongly associated with insulin resistance [45]. This location-dependent link may be due to environmental or genetic factors but may also be explained by the unique biochemical attributes of intra-abdominal adipose tissue [45].

One of the main functions of adipose tissue, particularly subcutaneous adipose tissue (SAT), is to store energy [54]. In adults, the number of fat cells in the body is relatively constant; however, an increase in body mass can result in an increase in either adipocyte number (hyperplasia) or adipocyte size (hypertrophy) [55]. Excess caloric intake, paired with a sedentary lifestyle, results in impaired adipogenesis or an inability to generate new fat cells [54]. When this process of hyperplasia is blunted, hypertrophy of SAT occurs instead [54].

During hypertrophy, cells may expand to the point where there is not enough vasculature to supply oxygen to the growing fat cells [54]. Combined with inadequate angiogenesis (development of new blood vessels), adipocyte hypoxia can occur and reactive oxygen species form [54]. Oxidative stress can directly affect endocrine and immune responses in the body [54]. Hypertrophic adipocytes also increase circulating triglycerides [56]. When adipose tissue grows, it may exceed its capacity to store more fat, causing additional free fatty acids (FFA) to be released into the bloodstream [54–57]. One of the major consequences is ectopic fat deposition in vital organs (i.e., muscle, heart, liver, etc.) [57] and the abdominal region, forming a visceral fat depot [58].

Intra-abdominal fat, also known as visceral adipose tissue (VAT), has many unique features distinct from subcutaneous adipose tissue (SAT) [59]. It is important to establish that this type of adipose tissue is more than an inert storage organ [54]. Visceral fat is highly vascularized, metabolically active, and home to an array of inflammatory cells [60]. Therefore, VAT plays a significant role in the secretion of adipokines and other endocrine-signaling molecules [60]. This hormonal activity has substantial metabolic implications as visceral fat delivers a large amount of FFA to the liver through the portal vein [58]. Abnormally high FFA levels in the liver result in metabolic changes that hinder insulin action and impair carbohydrate oxidation [58]. Combined with inflammation, oxidative stress, and other endocrine abnormalities associated with visceral fat, insulin resistance may rapidly develop [61].

While the mechanistic links between abdominal fat and insulin resistance are far from complete, it is well-established that the amount of total body fat is a significant but relatively small contributing factor [57]. The metabolic function and abdominal distribution of adipose tissue has much larger, far-reaching health implications that are still only partially understood.

The present study had several limitations. First, due to the cross-sectional nature of the investigation, there were temporal biases, and causal conclusions cannot be inferred. Because observational studies cannot control for all possible confounding variables, only associations can be identified between variables. Additionally, some participants had missing data which could increase the risk of bias in the survey estimates. Only subjects with complete data were included in the study. The investigation was also subject to selection/survival biases. Further, self-reported methods were used to assess sitting time and physical activity; thus, error in self-reporting could potentially influence results. However, error variance typically weakens relationships. It rarely strengthens them. Finally, NHANES data for 2019–2020 and after were not available due to COVID-19.

This investigation also exhibited many strengths. First, the present study used a large sample of 6931 U.S. adults representing multiple racial groups, including Mexican Americans, non-Hispanic (NH) Blacks, NH Whites, NH Asians, other Hispanics, and other races/multiraces. Second, subjects were selected at random using a unique, four-stage sampling strategy employed by NHANES, making the sample representative of and the results generalizable to the noninstitutionalized U.S. adult population aged 20–79. Third, multiple potential confounding variables were controlled during statistical analyses to minimize their influence on the association between sitting time and insulin resistance. Controlling these covariates also highlighted the extent to which this relationship is mediated by waist circumference, which has not been commonly studied in other investigations focusing on sitting time and insulin resistance. Fourth, insulin resistance was objectively indexed by HOMA-IR using fasting plasma and glucose samples taken by well-trained technicians.

## 5. Conclusion

In conclusion, findings from the present investigation demonstrate that a significant and linear relationship exists between sitting time and insulin resistance in U.S. adults. The association remained unchanged after controlling for demographic variables, cigarette smoking, and physical activity. Although adding BMI to the model weakened the relationship between sitting time and insulin resistance, it remained statistically significant. Conversely, replacing BMI with waist circumference in the model completely nullified the relationship, indicating that waist circumference was a powerful mediating variable. Effect modification confirmed these results. These findings emphasize not only the importance of minimizing sitting time but also the value of managing abdominal adiposity to reduce the risk of insulin resistance and other chronic diseases.

## Figures and Tables

**Table 1:** Percentile values of the continuous variables representing men and women of the United States (*N* = 6931).

Variable	IQR	Percentile				
		10th	25th	50th	75th	90th
Age (years)	25.9	23.9	31.2	43.9	57.1	66.4
Smoking (cigarettes per day)	0	0	0	0	0	9.4
HOMA-IR	2.47	0.93	1.41	2.31	3.88	6.19
L-HOMA-IR	0.44	-0.03	0.15	0.36	0.59	0.79
Physical activity (min MVPA per wk)	236.9	0	0	57.4	236.9	471.1
Body mass index (kg/m^2^)	8.3	21.7	24.1	27.8	32.4	37.7
Waist circumference (cm)	21.1	79.7	87.3	97.2	108.4	120.1
Sitting time (min per day)	258.7	114.3	215.4	338.6	474.1	597.4

IQR: interquartile range; HOMA-IR: homeostatic model assessment for insulin resistance; L-HOMA-IR: natural log of the HOMA-IR values; MVPA: number of minutes spent in moderate to vigorous physical activity per week. Table values include person-level weighted adjustments based on the sampling methods used by NHANES, so values are representative of the U.S. adult population.

**Table 2:** Mean differences in L-HOMA-IR across the three categories of sitting time in U.S. men and women, after adjusting for the covariates.

Criterion variable: L-HOMA-IR	Model	Low mean ± SE	Moderate mean ± SE	High mean ± SE	*F*	*p*
Covariates						
Demographics	1	0.37^a^ ± 0.008	0.40^b^ ± 0.012	0.43^c^ ± 0.012	12.6	<0.0001
Demographics + smoking	2	0.37^a^ ± 0.008	0.40^b^ ± 0.012	0.43^c^ ± 0.012	12.0	<0.0001
Demographics + activity	3	0.38^a^ ± 0.010	0.40^b^ ± 0.013	0.43^c^ ± 0.012	10.5	<0.0001
Demographics + BMI	4	0.40^a^ ± 0.007	0.41^a,b^ ± 0.009	0.42^b^ ± 0.009	3.7	0.0293
Demographics + WC	5	0.40^a^ ± 0.007	0.41^a^ ± 0.009	0.41^a^ ± 0.008	1.1	0.3349
All covariates	6	0.40^a^ ± 0.008	0.41^a^ ± 0.010	0.41^a^ ± 0.009	1.0	0.3881

L-HOMA-IR: natural log of HOMA-IR (homeostatic model assessment for insulin resistance); SE: standard error of the L-HOMA-IR mean; WC: waist circumference. The demographic covariates were age, sex, race, and year of assessment. Means with the same superscript letter (i.e., a, b, and c) on the same row did not differ significantly from each other. Model 1 adjusted for differences in the demographic variables only. Model 2 controlled for the model 1 covariates plus cigarette smoking. Model 3 controlled for the model 1 covariates plus physical activity. Model 4 adjusted for the model 1 covariates plus BMI. Model 5 adjusted for the model 1 covariates plus waist circumference. Model 6 adjusted for all the covariates simultaneously. Sitting time was divided into tertiles. However, because the tertile cut points included many participants, *N* differed across the 3 sitting time categories (low: *N* = 2483, moderate: *N* = 1868, and high: *N* = 2580). These frequencies are listed as weighted values due to the person-level weighted adjustments used by NHANES.

**Table 3:** Effect modification showing differences in mean L-HOMA-IR across the sitting time categories within individual waist circumference tertiles (*N* = 6931).

Waist circumference category	Sitting time category			*F*	*p*
	Low mean ± SE	Moderate mean ± SE	High mean ± SE		
Lowest tertile only (*N* = 2303)	0.159 ± 0.016	0.161 ± 0.018	0.174 ± 0.019	0.7	0.5022
Middle tertile only (*N* = 2320)	0.419 ± 0.019	0.412 ± 0.016	0.410 ± 0.020	0.2	0.8502
Highest tertile only (*N* = 2308)	0.649 ± 0.019	0.667 ± 0.020	0.672 ± 0.020	0.2	0.2144

L-HOMA-IR: natural log of HOMA-IR (homeostatic model assessment for insulin resistance); SE: standard error of the mean. None of the means on the same row were statistically different from each other. The means were adjusted for differences in the covariates: age, sex, race, year of assessment, BMI, physical activity, cigarette smoking, and waist circumference. Group frequencies are weighted values due to the person-level weighted adjustments used by NHANES.

## Data Availability

All the data supporting the reported results can be found online as part of the National Health and Nutrition Examination Survey (NHANES). The data are free and can be found at the following website: https://wwwn.cdc.gov/nchs/nhanes/Default.aspx
